# Supplementary material for: Are trials of psychological and psychosocial interventions for schizophrenia and psychosis included in the NICE guidelines pragmatic? A systematic review
Source: PLoS One. 2019 Sep 24;14(9):e0222891. doi: 10.1371/journal.pone.0222891 (PMC6759154; doi:10.1371/journal.pone.0222891)
Supplement: S1 Appendix — (DOCX) [file pone.0222891.s001.docx]

**S1_APPENDIX: REFERENCES OF INCLUDED STUDIES**

1. Atkinson JM, Coia DA, Gilmour WH, Harper JP. The Impact of Education Groups for People with Schizophrenia on Social Functioning and Quality of Life. British Journal of Psychiatry. 2018;168(2):199-204.

2. Bach P, Hayes SC. The use of acceptance and commitment therapy to prevent the rehospitalization of psychotic patients: A randomized controlled trial. Journal of Consulting and Clinical Psychology. 2002;70(5):1129-39.

3. Barrowclough C, Haddock G, Lobban F, Jones S, Siddle R, Roberts C, et al. Group cognitive-behavioural therapy for schizophrenia. Randomised controlled trial. The British journal of psychiatry : the journal of mental science. 2006;189:527-32.

4. Bauml J, Pitschel-Walz G, Volz A, Engel RR, Kessling W. Psychoeducation in schizophrenia: 7-year follow-up concerning rehospitalization and days in hospital in the Munich Psychosis Information Project Study. The Journal of clinical psychiatry. 2007;68(6):854-61.

5. Bechdolf A, Knost B, Kuntermann C, Schiller S, Klosterkotter J, Hambrecht M, et al. A randomized comparison of group cognitive-behavioural therapy and group psychoeducation in patients with schizophrenia. Acta Psychiatr Scand. 2004;110(1):21-8.

6. Bellack AS, Turner SM, Hersen M, Luber RF. An examination of the efficacy of social skills training for chronic schizophrenic patients. Hosp Community Psychiatry. 1984;35(10):1023-8.

7. Bellucci DM, Glaberman K, Haslam N. Computer-assisted cognitive rehabilitation reduces negative symptoms in the severely mentally ill. Schizophrenia research. 2003;59(2-3):225-32.

8. Benedict RH, Harris AE, Markow T, McCormick JA, Nuechterlein KH, Asarnow RF. Effects of attention training on information processing in schizophrenia. Schizophr Bull. 1994;20(3):537-46.

9. Bloch S, Szmukler GI, Herrman H, Benson A, Colussa S. Counseling caregivers of relatives with schizophrenia: themes, interventions, and caveats. Fam Process. 1995;34(4):413-25.

10. Bradley GM, Couchman GM, Perlesz A, Nguyen AT, Singh B, Riess C. Multiple-family group treatment for English- and Vietnamese-speaking families living with schizophrenia. Psychiatr Serv. 2006;57(4):521-30.

11. Bradshaw. Structured Group Work for Individuals with Schizophrenia: A Coping Skills Approach. Research on Social Work Practice. 1996;6:139-54.

12. Bradshaw W. Integrating cognitive-behavioral psychotherapy for persons with schizophrenia into a psychiatric rehabilitation program: results of a three year trial. Community Ment Health J. 2000;36(5):491-500.

13. Bressi C, Manenti S, Frongia P, Porcellana M, Invernizzi G. Systemic family therapy in schizophrenia: a randomized clinical trial of effectiveness. Psychother Psychosom. 2008;77(1):43-9.

14. Brown MA, Munford AM. Life skills training for chronic schizophrenics. The Journal of nervous and mental disease. 1983;171(8):466-70.

15. Buchkremer G, Monking HS, Holle R, Hornung W. The impact of therapeutic relatives' groups on the course of illness of schizophrenic patients. Eur Psychiatry. 1995;10(1):17-27.

16. Burda TWS, Florentine Dominguez, and Vivian Vera. Computer-Assisted Cognitive Rehabilitation of Chronic Psychiatric Inpatients. Computers in Human Behavior. 1994;10(3):359-68.

17. Carra G, Montomoli C, Clerici M, Cazzullo CL. Family interventions for schizophrenia in Italy: randomized controlled trial. European archives of psychiatry and clinical neuroscience. 2007;257(1):23-30.

18. Cather C, Penn D, Otto MW, Yovel I, Mueser KT, Goff DC. A pilot study of functional Cognitive Behavioral Therapy (fCBT) for schizophrenia. Schizophrenia research. 2005;74(2-3):201-9.

19. Chabannes JP, Bazin N, Leguay D, Nuss P, Peretti CS, Tatu P, et al. Two-year study of relapse prevention by a new education program in schizophrenic patients treated with the same antipsychotic drug. Eur Psychiatry. 2008;23(1):8-13.

20. Chan SH, Lee SW, Chan IW. TRIP: a psycho-educational programme in Hong Kong for people with schizophrenia. Occup Ther Int. 2007;14(2):86-98.

21. Cheng LY, Chan S. Psychoeducation program for chinese family carers of members with schizophrenia. West J Nurs Res. 2005;27(5):583-99; comment 600-3.

22. Chien H-C, Ku C-H, Lu R-B, Chu H, Tao Y-H, Chou K-R. Effects of social skills training on improving social skills of patients with schizophrenia. Archives of Psychiatric Nursing. 2003;17(5):228-36.

23. Chien WT, Chan SW. One-year follow-up of a multiple-family-group intervention for Chinese families of patients with schizophrenia. Psychiatr Serv. 2004;55(11):1276-84.

24. Chien WT, Norman I, Thompson DR. A randomized controlled trial of a mutual support group for family caregivers of patients with schizophrenia. Int J Nurs Stud. 2004;41(6):637-49.

25. Chien WT, Wong KF. A family psychoeducation group program for chinese people with schizophrenia in Hong Kong. Psychiatr Serv. 2007;58(7):1003-6.

26. Choi KH, Kwon JH. Social cognition enhancement training for schizophrenia: a preliminary randomized controlled trial. Community Ment Health J. 2006;42(2):177-87.

27. Cunningham Owens DG, Carroll A, Fattah S, Clyde Z, Coffey I, Johnstone EC. A randomized, controlled trial of a brief interventional package for schizophrenic out-patients. Acta Psychiatr Scand. 2001;103(5):362-9.

28. Daniels L. A group cognitive-behavioral and process-oriented approach to treating the social impairment and negative symptoms associated with chronic mental illness. J Psychother Pract Res. 1998;7(2):167-76.

29. Dobson DJ, McDougall G, Busheikin J, Aldous J. Effects of social skills training and social milieu treatment on symptoms of schizophrenia. Psychiatr Serv. 1995;46(4):376-80.

30. Donlon PT, Rada RT, Knight SW. A therapeutic aftercare setting for "refractory" chronic schizophrenic patients. Am J Psychiatry. 1973;130(6):682-4.

31. Drury V, Birchwood M, Cochrane R, Macmillan F. Cognitive therapy and recovery from acute psychosis: a controlled trial. I. Impact on psychotic symptoms. The British journal of psychiatry : the journal of mental science. 1996;169(5):593-601.

32. Durham RC, Guthrie M, Morton RV, Reid DA, Treliving LR, Fowler D, et al. Tayside-Fife clinical trial of cognitive-behavioural therapy for medication-resistant psychotic symptoms. Results to 3-month follow-up. The British journal of psychiatry : the journal of mental science. 2003;182:303-11.

33. Dyck DG, Short RA, Hendryx MS, Norell D, Myers M, Patterson T, et al. Management of negative symptoms among patients with schizophrenia attending multiple-family groups. Psychiatr Serv. 2000;51(4):513-9.

34. Eack SM, Hogarty GE, Greenwald DP, Hogarty SS, Keshavan MS. Cognitive enhancement therapy improves emotional intelligence in early course schizophrenia: preliminary effects. Schizophrenia research. 2007;89(1-3):308-11.

35. Eckman TA, Wirshing WC, Marder SR, Liberman RP, Johnston-Cronk K, Zimmermann K, et al. Technique for training schizophrenic patients in illness self-management: a controlled trial. Am J Psychiatry. 1992;149(11):1549-55.

36. England M. Efficacy of cognitive nursing intervention for voice hearing. Perspect Psychiatr Care. 2007;43(2):69-76.

37. Falloon IR, Boyd JL, McGill CW, Razani J, Moss HB, Gilderman AM. Family management in the prevention of exacerbations of schizophrenia: a controlled study. N Engl J Med. 1982;306(24):1437-40.

38. Garety PA, Fowler DG, Freeman D, Bebbington P, Dunn G, Kuipers E. Cognitive--behavioural therapy and family intervention for relapse prevention and symptom reduction in psychosis: randomised controlled trial. The British journal of psychiatry : the journal of mental science. 2008;192(6):412-23.

39. Glynn ETR, Spencer Eth, George G. Paz, Gregory B. Leong, Andrew L. Shaner, and Walter Van Vort. Schizophrenic Symptoms, Work Adjustment, and Behavioral Family Therapy. REHABILITATION PSYCHOLOGY. 1992;34:4.

40. Glynn SM, Marder SR, Liberman RP, Blair K, Wirshing WC, Wirshing DA, et al. Supplementing clinic-based skills training with manual-based community support sessions: effects on social adjustment of patients with schizophrenia. Am J Psychiatry. 2002;159(5):829-37.

41. Goldstein MJ, Rodnick EH, Evans JR, May PR, Steinberg MR. Drug and family therapy in the aftercare of acute schizophrenics. Arch Gen Psychiatry. 1978;35(10):1169-77.

42. Granholm E, McQuaid JR, McClure FS, Auslander LA, Perivoliotis D, Pedrelli P, et al. A randomized, controlled trial of cognitive behavioral social skills training for middle-aged and older outpatients with chronic schizophrenia. Am J Psychiatry. 2005;162(3):520-9.

43. Gray R, Leese M, Bindman J, Becker T, Burti L, David A, et al. Adherence therapy for people with schizophrenia. European multicentre randomised controlled trial. The British journal of psychiatry : the journal of mental science. 2006;189:508-14.

44. Green BL, Wehling C, Taisky GJ. Group Art Therapy as an Adjunct to Treatment for Chronic Outpatients. Psychiatric Services. 1987;38(9):988-91.

45. Gumley A, O'Grady M, McNay L, Reilly J, Power K, Norrie J. Early intervention for relapse in schizophrenia: results of a 12-month randomized controlled trial of cognitive behavioural therapy. Psychological Medicine. 2003;33(3):419-31.

46. GUNDERSON MD, a AND ARLENE F. FRANK,. Effects of Psychotherapy in Schizophrenia. THE YALE JOURNAL OF BIOLOGY AND MEDICINE. 1985;58.

47. Hadas-Lidor NK, S Tyano, Weizman. Effectiveness of dynamic cognitive intervention in rehabilitation of clients with schizophrenia. Clinical Rehabilitation. 2001;15:349–59.

48. Haddock G, Tarrier N, Morrison AP, Hopkins R, Drake R, Lewis S. A pilot study evaluating the effectiveness of individual inpatient cognitive-behavioural therapy in early psychosis. Soc Psychiatry Psychiatr Epidemiol. 1999;34(5):254-8.

49. Hayashi N, Yamashina M, Igarashi Y, Kazamatsuri H. Improvement of patient attitude toward treatment among inpatients with schizophrenia and its related factors: controlled study of a psychological approach. Compr Psychiatry. 2001;42(3):240-6.

50. HAYES WKH, FRANCIS T. VARGHESE. Social Skills Training With Chronic Schizophrenic Patients: Effects on Negative Symptoms and Community Functioning

BEHAVIORAL THERAP. 1995;26:433-49.

51. Herz MI, Lamberti JS, Mintz J, Scott R, O'Dell SP, McCartan L, et al. A program for relapse prevention in schizophrenia: a controlled study. Arch Gen Psychiatry. 2000;57(3):277-83.

52. Hogarty GE, Flesher S, Ulrich R, Carter M, Greenwald D, Pogue-Geile M, et al. Cognitive enhancement therapy for schizophrenia: effects of a 2-year randomized trial on cognition and behavior. Arch Gen Psychiatry. 2004;61(9):866-76.

53. Hogarty GE, Greenwald D, Ulrich RF, Kornblith SJ, DiBarry AL, Cooley S, et al. Three-year trials of personal therapy among schizophrenic patients living with or independent of family, II: Effects on adjustment of patients. Am J Psychiatry. 1997;154(11):1514-24.

54. Hogarty GE, Kornblith SJ, Greenwald D, DiBarry AL, Cooley S, Ulrich RF, et al. Three-year trials of personal therapy among schizophrenic patients living with or independent of family, I: Description of study and effects on relapse rates. Am J Psychiatry. 1997;154(11):1504-13.

55. Hornung WP, Feldmann R, Klingberg S, Buchkremer G, Reker T. Long-term effects of a psychoeducational psychotherapeutic intervention for schizophrenic outpatients and their key-persons--results of a five-year follow-up. European archives of psychiatry and clinical neuroscience. 1999;249(3):162-7.

56. Jackson H, McGorry P, Edwards J, Hulbert C, Henry L, Harrigan S, et al. A controlled trial of cognitively oriented psychotherapy for early psychosis (COPE) with four-year follow-up readmission data. Psychol Med. 2005;35(9):1295-306.

57. Jackson HJ, McGorry PD, Killackey E, Bendall S, Allott K, Dudgeon P, et al. Acute-phase and 1-year follow-up results of a randomized controlled trial of CBT versus Befriending for first-episode psychosis: the ACE project. Psychol Med. 2008;38(5):725-35.

58. Jenner JA, Nienhuis FJ, Wiersma D, van de Willige G. Hallucination focused integrative treatment: a randomized controlled trial. Schizophr Bull. 2004;30(1):133-45.

59. Jones RB, Atkinson JM, Coia DA, Paterson L, Morton AR, McKenna K, et al. Randomised trial of personalised computer based information for patients with schizophrenia. BMJ. 2001;322(7290):835-40.

60. Kemp R, Hayward P, Applewhaite G, Everitt B, David A. Compliance therapy in psychotic patients: randomised controlled trial. BMJ. 1996;312(7027):345-9.

61. Kopelowicz A, Zarate R, Gonzalez Smith V, Mintz J, Liberman RP. Disease management in Latinos with schizophrenia: a family-assisted, skills training approach. Schizophr Bull. 2003;29(2):211-27.

62. Kuipers E, Garety P, Fowler D, Dunn G, Bebbington P, Freeman D, et al. London–East Anglia randomised controlled trial of cognitive–behavioural therapy for psychosis. British Journal of Psychiatry. 2018;171(4):319-27.

63. Kurtz MM, Seltzer JC, Shagan DS, Thime WR, Wexler BE. Computer-assisted cognitive remediation in schizophrenia: what is the active ingredient? Schizophrenia research. 2007;89(1-3):251-60.

64. Leavey G, Gulamhussein S, Papadopoulos C, Johnson-Sabine E, Blizard BOB, King M. A randomized controlled trial of a brief intervention for families of patients with a first episode of psychosis. Psychological Medicine. 2004;34(3):423-31.

65. Leclerc C, Lesage AD, Ricard N, Lecomte T, Cyr M. Assessment of a new rehabilitative coping skills module for persons with schizophrenia. Am J Orthopsychiatry. 2000;70(3):380-8.

66. Lecomte T, Leclerc C, Corbiere M, Wykes T, Wallace CJ, Spidel A. Group cognitive behavior therapy or social skills training for individuals with a recent onset of psychosis? Results of a randomized controlled trial. The Journal of nervous and mental disease. 2008;196(12):866-75.

67. Leff J, Berkowitz R, Shavit N, Strachan A, Glass I, Vaughn C. A trial of family therapy v. a relatives group for schizophrenia. The British journal of psychiatry : the journal of mental science. 1989;154:58-66.

68. Leff J, Kuipers L, Berkowitz R, Eberlein-Vries R, Sturgeon D. A controlled trial of social intervention in the families of schizophrenic patients. The British journal of psychiatry : the journal of mental science. 1982;141:121-34.

69. Levine. Cognitive group therapy for paronoid schizophrenics: applying cognitive dissonance. journal of cognitive psychotherapy 1998;12(1).

70. Lewis S, Tarrier N, Haddock G, Bentall R, Kinderman P, Kingdon D, et al. Randomised controlled trial of cognitive-behavioural therapy in early schizophrenia: Acute-phase outcomes. British Journal of Psychiatry. 2018;181(S43):s91-s7.

71. Li Z, Arthur D. Family education for people with schizophrenia in Beijing, China: randomised controlled trial. The British journal of psychiatry : the journal of mental science. 2005;187:339-45.

72. Liberman RP, Wallace CJ, Blackwell G, Kopelowicz A, Vaccaro JV, Mintz J. Skills training versus psychosocial occupational therapy for persons with persistent schizophrenia. Am J Psychiatry. 1998;155(8):1087-91.

73. Littrell KH, Hilligoss NM, Kirshner CD, Petty RG, Johnson CG. The effects of an educational intervention on antipsychotic-induced weight gain. J Nurs Scholarsh. 2003;35(3):237-41.

74. Lukotf CJW, Robert P. Llberman, and Karen Burke. A Holistic Program for Chronic Schizophrenic Patients. Schizophr Bull. 1986;12(2):276.

75. MACPHERSONB I. A Controlled Study of Education About Drug Treatment in Schizophrenia. British Journal of Psychiatry. 1996;168:709-17.

76. Magliano L, Fiorillo A, Malangone C, De Rosa C, Maj M. Patient Functioning and Family Burden in a Controlled, Real-World Trial of Family Psychoeducation for Schizophrenia. Psychiatric Services. 2006;57(12):1784-91.

77. Maneesakorn S, Robson D, Gournay K, Gray R. An RCT of adherence therapy for people with schizophrenia in Chiang Mai, Thailand. Journal of Clinical Nursing. 2007;0(0):070621074500047-???

78. Marder SR, wirshing. Mintz, Mckenzie, johnston, eckman, lebell, zimmerman, liberman 2 years outcom of social skills trianind and group psychotherapy for outpatient with schizophrenia am j psych. 1996;153(12).

79. May PR, Tuma AH, Yale C, Potepan P, Dixon WJ. Schizophrenia--a follow-up study of results of treatment. Arch Gen Psychiatry. 1976;33(4):481-6.

80. McFARLANE MDaBL, PH.D.a ROBERT DUSHAY, PH.D.a JOANNE MARCHAL, M.A.b JOHN CRILLY, C.S.W.a. Psychoeducational Multiple Family Groups: Four-Year Relapse Outcome in Schizophrenia. Fam Proc 1995;34:127-44.

81. McFarlane WR, Lukens E, Link B, Dushay R, Deakins SA, Newmark M, et al. Multiple-family groups and psychoeducation in the treatment of schizophrenia. Arch Gen Psychiatry. 1995;52(8):679-87.

82. McLeod T, Morris M, Birchwood M, Dovey A. Cognitive behavioural therapy group work with voice hearers. Part 1. Br J Nurs. 2007;16(4):248-52.

83. McLeod T, Morris M, Birchwood M, Dovey A. Cognitive behavioural therapy group work with voice hearers. Part 2. Br J Nurs. 2007;16(5):292-5.

84. Medalia A, Aluma M, Tryon W, Merriam AE. Effectiveness of attention training in schizophrenia. Schizophr Bull. 1998;24(1):147-52.

85. Medalia A, Revheim N, Casey M. Remediation of memory disorders in schizophrenia. Psychol Med. 2000;30(6):1451-9.

86. Merinder LB, Viuff AG, Laugesen HD, Clemmensen K, Misfelt S, Espensen B. Patient and relative education in community psychiatry: a randomized controlled trial regarding its effectiveness. Soc Psychiatry Psychiatr Epidemiol. 1999;34(6):287-94.

87. Montero I, Asencio A, Hernandez I, Masanet MJ, Lacruz M, Bellver F, et al. Two strategies for family intervention in schizophrenia: a randomized trial in a Mediterranean environment. Schizophr Bull. 2001;27(4):661-70.

88. Ng C. social skills training in hong kong chinese patient with chronic schizophrenia hong kong journal of psychiatry 2006;16.

89. Nitsun M, Stapleton JH, Bender MP. Movement and drama therapy with long-stay schizophrenics. Br J Med Psychol. 1974;47(2):101-19.

90. Nugter A, Dingemans P, Van der Does JW, Linszen D, Gersons B. Family treatment, expressed emotion and relapse in recent onset schizophrenia. Psychiatry research. 1997;72(1):23-31.

91. O'Brien CP, Hamm KB, Ray BA, Pierce JF, Luborsky L, Mintz J. Group vs individual psychotherapy with schizophrenics. A controlled outcome study. Arch Gen Psychiatry. 1972;27(4):474-8.

92. O'Donnell C, Donohoe G, Sharkey L, Owens N, Migone M, Harries R, et al. Compliance therapy: a randomised controlled trial in schizophrenia. BMJ. 2003;327(7419):834.

93. Patterson TL, Mausbach BT, McKibbin C, Goldman S, Bucardo J, Jeste DV. Functional adaptation skills training (FAST): a randomized trial of a psychosocial intervention for middle-aged and older patients with chronic psychotic disorders. Schizophrenia research. 2006;86(1-3):291-9.

94. Pelc LaI. A Cognitive-Behavioral Program to Improve Compliance with Medication in Patients with Schizophrenia. Int J Ment Health 1996;25(1):51-6.

95. Penades R, Catalan R, Salamero M, Boget T, Puig O, Guarch J, et al. Cognitive remediation therapy for outpatients with chronic schizophrenia: a controlled and randomized study. Schizophrenia research. 2006;87(1-3):323-31.

96. Peniston. group assetion and contingent time-out procedures in the control of assaultive behaviours in schizphrenic medical psychoterapy. 1988;1:131-41.

97. Pinto A, La Pia S, Mennella R, Giorgio D, DeSimone L. Cognitive-behavioral therapy and clozapine for clients with treatment-refractory schizophrenia. Psychiatr Serv. 1999;50(7):901-4.

98. Posner CM, Wilson KG, Kral MJ, Lander S, McIlwraith RD. Family psychoeducational support groups in schizophrenia. Am J Orthopsychiatry. 1992;62(2):206-18.

99. Ran MS, Xiang MZ, Chan CL, Leff J, Simpson P, Huang MS, et al. Effectiveness of psychoeducational intervention for rural Chinese families experiencing schizophrenia--a randomised controlled trial. Soc Psychiatry Psychiatr Epidemiol. 2003;38(2):69-75.

100. Rector NA, Seeman MV, Segal ZV. Cognitive therapy for schizophrenia: a preliminary randomized controlled trial. Schizophrenia research. 2003;63(1-2):1-11.

101. Richardson P, Jones K, Evans C, Stevens P, Rowe A. Exploratory RCT of art therapy as an adjunctive treatment in schizophrenia. Journal of Mental Health. 2009;16(4):483-91.

102. Rohricht F, Priebe S. Effect of body-oriented psychological therapy on negative symptoms in schizophrenia: a randomized controlled trial. Psychol Med. 2006;36(5):669-78.

103. Roncone R, Mazza M, Frangou I, De Risio A, Ussorio D, Tozzini C, et al. Rehabilitation of theory of mind deficit in schizophrenia: a pilot study of metacognitive strategies in group treatment. Neuropsychological Rehabilitation. 2004;14(4):421-35.

104. Sartory G, Zorn C, Groetzinger G, Windgassen K. Computerized cognitive remediation improves verbal learning and processing speed in schizophrenia. Schizophrenia research. 2005;75(2-3):219-23.

105. Schooler NR, Keith SJ, Severe JB, Matthews SM, Bellack AS, Glick ID, et al. Relapse and rehospitalization during maintenance treatment of schizophrenia. The effects of dose reduction and family treatment. Arch Gen Psychiatry. 1997;54(5):453-63.

106. Sensky T, Turkington D, Kingdon D, Scott JL, Scott J, Siddle R, et al. A Randomized Controlled Trial of Cognitive-Behavioral Therapy for Persistent Symptoms in Schizophrenia Resistant to Medication. Archives of General Psychiatry. 2000;57(2).

107. Shin SK, Lukens EP. Effects of psychoeducation for Korean Americans with chronic mental illness. Psychiatr Serv. 2002;53(9):1125-31.

108. Sibitz I, Amering M, Gossler R, Unger A, Katschnig H. One-year outcome of low-intensity booster sessions versus care as usual in psychosis patients after a short-term psychoeducational intervention. Eur Psychiatry. 2007;22(4):203-10.

109. Silverstein SM, Hatashita-Wong M, Solak BA, Uhlhaas P, Landa Y, Wilkniss SM, et al. Effectiveness of a two-phase cognitive rehabilitation intervention for severely impaired schizophrenia patients. Psychological Medicine. 2004;35(6):829-37.

110. Smith JV, Birchwood MJ. Specific and non-specific effects of educational intervention with families living with a schizophrenic relative. The British journal of psychiatry : the journal of mental science. 1987;150:645-52.

111. So C, Chang, Hung, Chan effects of brief intervention for carers of people with first-episode psychosis: a waiting-list controlled study hong kong journal of psychiatry. 2006;16:92-100.

112. Spaulding WD, Reed D, Sullivan M, Richardson C, Weiler M. Effects of cognitive treatment in psychiatric rehabilitation. Schizophr Bull. 1999;25(4):657-76.

113. Stanton AH, Gunderson JG, Knapp PH, Frank AF, Vannicelli ML, Schnitzer R, et al. Effects of psychotherapy in schizophrenia: I. Design and implementation of a controlled study. Schizophr Bull. 1984;10(4):520-63.

114. Startup M, Jackson MC, Bendix S. North Wales randomized controlled trial of cognitive behaviour therapy for acute schizophrenia spectrum disorders: outcomes at 6 and 12 months. Psychological Medicine. 2004;34(3):413-22.

115. Szmukler G, Kuipers E, Joyce J, Harris T, Leese M, Maphosa W, et al. An exploratory randomised controlled trial of a support programme for carers of patients with a psychosis. Soc Psychiatry Psychiatr Epidemiol. 2003;38(8):411-8.

116. Talwar N, Crawford MJ, Maratos A, Nur U, McDermott O, Procter S. Music therapy for in-patients with schizophrenia: exploratory randomised controlled trial. The British journal of psychiatry : the journal of mental science. 2006;189:405-9.

117. Tarrier N, Yusupoff L, Kinney C, McCarthy E, Gledhill A, Haddock G, et al. Randomised controlled trial of intensive cognitive behaviour therapy for patients with chronic schizophrenia. BMJ. 1998;317(7154):303-7.

118. Trower P, Birchwood M, Meaden A, Byrne S, Nelson A, Ross K. Cognitive therapy for command hallucinations: randomised controlled trial. The British journal of psychiatry : the journal of mental science. 2004;184:312-20.

119. tsang w. the effects of a compliance therapy programme on chinese male patients with schizophrenia AJNS. 2005;8(2):47.

120. Turkington D, Kingdon D, Turner T, Insight into Schizophrenia Research G. Effectiveness of a brief cognitive-behavioural therapy intervention in the treatment of schizophrenia. The British journal of psychiatry : the journal of mental science. 2002;180:523-7.

121. Twamley EW, Savla GN, Zurhellen CH, Heaton RK, Jeste DV. Development and Pilot Testing of a Novel Compensatory Cognitive Training Intervention for People with Psychosis. Am J Psychiatr Rehabil. 2008;11(2):144-63.

122. Ucok A, Cakir S, Duman ZC, Discigil A, Kandemir P, Atli H. Cognitive predictors of skill acquisition on social problem solving in patients with schizophrenia. European archives of psychiatry and clinical neuroscience. 2006;256(6):388-94.

123. Ulrich G, Houtmans T, Gold C. The additional therapeutic effect of group music therapy for schizophrenic patients: a randomized study. Acta Psychiatr Scand. 2007;116(5):362-70.

124. Valencia M, Rascon ML, Juarez F, Murow E. A psychosocial skills training approach in Mexican out-patients with schizophrenia. Psychol Med. 2007;37(10):1393-402.

125. Valmaggia LR, van der Gaag M, Tarrier N, Pijnenborg M, Slooff CJ. Cognitive-behavioural therapy for refractory psychotic symptoms of schizophrenia resistant to atypical antipsychotic medication. Randomised controlled trial. The British journal of psychiatry : the journal of mental science. 2005;186:324-30.

126. van der Gaag M, Kern RS, van den Bosch RJ, Liberman RP. A controlled trial of cognitive remediation in schizophrenia. Schizophr Bull. 2002;28(1):167-76.

127. Vaughan K, Doyle M, McConaghy N, Blaszczynski A, Fox A, Tarrier N. The Sydney intervention trial: a controlled trial of relatives' counselling to reduce schizophrenic relapse. Soc Psychiatry Psychiatr Epidemiol. 1992;27(1):16-21.

128. Velligan CCB-T, Cindy Huntzinger, Janice Ritch, Natalie Ledbetter, Thomas J. Prihoda, Alexander L. Miller. Randomized Controlled Trial of the Use of Compensatory Strategies to Enhance Adaptive Functioning in Outpatients With Schizophrenia. am j psych. 2000;157:1317-23.

129. Velligan DI, Diamond PM, Maples NJ, Mintz J, Li X, Glahn DC, et al. Comparing the efficacy of interventions that use environmental supports to improve outcomes in patients with schizophrenia. Schizophrenia research. 2008;102(1-3):312-9.

130. Velligan DI, Diamond PM, Mintz J, Maples N, Li X, Zeber J, et al. The use of individually tailored environmental supports to improve medication adherence and outcomes in schizophrenia. Schizophr Bull. 2008;34(3):483-93.

131. Velligan TJP, Janice L. Ritch, Natalie Maples, C. Christine Bow'Thomas, and Albana Dassori. A Randomized Single-Blind Pilot Study of Compensatory Strategies in Schizophrenia Outpatients. Schizophrenia Bulletin. 2002;28(2):283-92.

132. Vollema MG, Geurtsen GJ, van Voorst AJ. Durable improvements in Wisconsin Card Sorting Test performance in schizophrenic patients. Schizophrenia research. 1995;16(3):209-15.

133. Vreeland B, Minsky S, Yanos PT, Menza M, Gara M, Kim E, et al. Efficacy of the team solutions program for educating patients about illness management and treatment. Psychiatr Serv. 2006;57(6):822-8.

134. Wallace CJ, Boone SE. Cognitive factors in the social skills of schizophrenic patients: implications for treatment. Nebr Symp Motiv. 1984;31:283-318.

135. Wykes T, Hayward P, Thomas N, Green N, Surguladze S, Fannon D, et al. What are the effects of group cognitive behaviour therapy for voices? A randomised control trial. Schizophrenia research. 2005;77(2-3):201-10.

136. Wykes T, Newton E, Landau S, Rice C, Thompson N, Frangou S. Cognitive remediation therapy (CRT) for young early onset patients with schizophrenia: an exploratory randomized controlled trial. Schizophrenia research. 2007;94(1-3):221-30.

137. Wykes T, Reeder C, Corner J, Williams C, Everitt B. The effects of neurocognitive remediation on executive processing in patients with schizophrenia. Schizophr Bull. 1999;25(2):291-307.

138. Wykes T, Reeder C, Landau S, Everitt B, Knapp M, Patel A, et al. Cognitive remediation therapy in schizophrenia: randomised controlled trial. The British journal of psychiatry : the journal of mental science. 2007;190:421-7.

139. Xiang Y, Weng Y, Li W, Gao L, Chen G, Xie L, et al. Training patients with schizophrenia with the community re-entry module: a controlled study. Soc Psychiatry Psychiatr Epidemiol. 2006;41(6):464-9.

140. Xiang YT, Weng YZ, Li WY, Gao L, Chen GL, Xie L, et al. Efficacy of the Community Re-Entry Module for patients with schizophrenia in Beijing, China: outcome at 2-year follow-up. The British journal of psychiatry : the journal of mental science. 2007;190:49-56.

141. Xiong W, Phillips MR, Hu X, Wang R, Dai Q, Kleinman J, et al. Family-based intervention for schizophrenic patients in China. A randomised controlled trial. The British journal of psychiatry : the journal of mental science. 1994;165(2):239-47.

142. YANG Cl, weng, ZHANG, MA BIO. PSYCHOSOCIAL REHABILITATION EFFECTS OF MUSIC THERAPY IN CHRONIC SCHIZOPHRENIA. hong kong journal of psychiatry. 1998;8(1):38-40.

143. Zhang M, Wang M, Li J, Phillips MR. Randomised-control trial of family intervention for 78 first-episode male schizophrenic patients. An 18-month study in Suzhou, Jiangsu. Br J Psychiatry Suppl. 1994(24):96-102
