# Supplementary material for: Are trials of psychological and psychosocial interventions for schizophrenia and psychosis included in the NICE guidelines pragmatic? A systematic review
Source: PLoS One. 2019 Sep 24;14(9):e0222891. doi: 10.1371/journal.pone.0222891 (PMC6759154; doi:10.1371/journal.pone.0222891)
Supplement: S1 Table — (DOCX) [file pone.0222891.s002.docx]

**S1_TABLE. TABLE OF MEAN SCORES AND CATEGORY OF INCLUDED STUDIES**

+-------------------------------------------------------------+

| STUDYID category mean score

|-------------------------------------------------------------|

1. | Atkinson1996 pragmatic_study 3.777778 |

2. | Bach2002 pragmatic_study 3.666667 |

3. | Barrowclough1999 pragmatic_study 3.666667 |

4. | Barrow-Clough2006 intermediate_study 3 |

5. | Bauml1996 pragmatic_study 4.111111 |

|-------------------------------------------------------------|

6. | Bechdolf2004 pragmatic_study 3.666667 |

7. | Bellack1984 intermediate_study 2.777778 |

8. | Bellucci2002 explanatory_study 2.444444 |

9. | Benedict 1994 explanatory_study 2.222222 |

10. | Bloch1995 explanatory_study 2.444444 |

|-------------------------------------------------------------|

11. | Bradley2006 intermediate_study 2.888889 |

12. | Bradshaw1996 intermediate_study 3.333333 |

13. | Bradshaw2000 pragmatic_study 3.888889 |

14. | Bressi2008 intermediate_study 3.222222 |

15. | Brown1983 explanatory_study 2 |

|-------------------------------------------------------------|

16. | Buchkremer1995 intermediate_study 3 |

17. | Burda 1994 intermediate_study 2.666667 |

18. | Carra2007 intermediate_study 3.111111 |

19. | Cather2005 explanatory_study 2.333333 |

20. | Chabannes2008 pragmatic_study 3.666667 |

|-------------------------------------------------------------|

21. | Chan2007a pragmatic_study 3.777778 |

22. | Cheng2005 intermediate_study 3.333333 |

23. | Chien2003 intermediate_study 2.777778 |

24. | Chien2004a pragmatic_study 3.888889 |

25. | Chien2004b pragmatic_study 4.555555 |

|-------------------------------------------------------------|

26. | Chien2007 pragmatic_study 3.666667 |

27. | Choi2006 explanatory_study 2.333333 |

28. | Cunninghamownes2001 pragmatic_study 3.555556 |

29. | Daniels1998 explanatory_study 2.222222 |

30. | Dobson1995 intermediate_study 2.777778 |

|-------------------------------------------------------------|

31. | Donlon 1973 intermediate_study 3 |

32. | Drury1996 intermediate_study 2.555556 |

33. | Durham2003 pragmatic_study 3.555556 |

34. | Dyck2000 intermediate_study 2.555556 |

35. | Eack 2007 intermediate_study 2.777778 |

|-------------------------------------------------------------|

36. | Eckmann1992 intermediate_study 2.555556 |

37. | England2007 pragmatic_study 3.555556 |

38. | Falloon1981 intermediate_study 3.222222 |

39. | Finch&Wallace 1977 intermediate_study 3.333333 |

40. | Garety2008 intermediate_study 2.555556 |

|-------------------------------------------------------------|

41. | Glynn1992 intermediate_study 3.444444 |

42. | Glynn2002 explanatory_study 2.333333 |

43. | Goldstein1978 intermediate_study 3.444444 |

44. | Granholm2005 explanatory_study 2.111111 |

45. | Gray 2006 pragmatic_study 3.888889 |

|-------------------------------------------------------------|

46. | Green1987 pragmatic_study 3.666667 |

47. | Gumley2003 pragmatic_study 3.666667 |

48. | Gunderson1984 explanatory_study 1.666667 |

49. | Hadas-lidor2001 intermediate_study 2.888889 |

50. | Haddock1999 intermediate_study 3.333333 |

|-------------------------------------------------------------|

51. | Hayashi2001 intermediate_study 3.222222 |

52. | Hayes1995 explanatory_study 2.222222 |

53. | Herz2000 explanatory_study 2.222222 |

54. | Hogarty1997 intermediate_study 3 |

55. | Hogarty2004 intermediate_study 3 |

|-------------------------------------------------------------|

56. | Hornung1995a pragmatic_study 3.555556 |

57. | Jackson2005 NOT RANDOMIZED explanatory_study 1 |

58. | Jackson2007 intermediate_study 3 |

59. | Jenner2004 pragmatic_study 3.666667 |

60. | Jones 2001 pragmatic_study 3.777778 |

|-------------------------------------------------------------|

61. | Kemp 1996 pragmatic_study 4.222222 |

62. | Kope1icz2003 intermediate_study 2.555556 |

63. | Kuipers1997 pragmatic_study 3.666667 |

64. | Kurtz 2007 intermediate_study 2.555556 |

65. | Leavey2004 pragmatic_study 4.333333 |

|-------------------------------------------------------------|

66. | Leclerc2000 intermediate_study 3.222222 |

67. | Lecompte1996 pragmatic_study 4.111111 |

68. | Lecomte2008 pragmatic_study 4.111111 |

69. | Leff1982 intermediate_study 3.444444 |

70. | Leff1989 intermediate_study 3.444444 |

|-------------------------------------------------------------|

71. | levine1998 intermediate_study 2.888889 |

72. | Lewis2002 pragmatic_study 4.222222 |

73. | Li2005 intermediate_study 3.222222 |

74. | Liberman1998 explanatory_study 2.444444 |

75. | Linszen1996 intermediate_study 3.111111 |

|-------------------------------------------------------------|

76. | Littrell 2003 intermediate_study 3.444444 |

77. | Lukoff1986 explanatory_study 2.444444 |

78. | Macpherson1996 intermediate_study 2.888889 |

79. | Magliano2006 pragmatic_study 3.666667 |

80. | Maneesakorn 2007 pragmatic_study 3.666667 |

|-------------------------------------------------------------|

81. | Marder1996 intermediate_study 2.555556 |

82. | May anno 1976 intermediate_study 2.666667 |

83. | Mcfarlane1995a pragmatic_study 3.666667 |

84. | Mcfarlane1995b intermediate_study 3.111111 |

85. | Mcleod2007 pragmatic_study 3.666667 |

|-------------------------------------------------------------|

86. | Medalia1998 intermediate_study 3.222222 |

87. | Medalia2000 intermediate_study 3.222222 |

88. | Merinder1999 intermediate_study 3.222222 |

89. | Montero2001 intermediate_study 2.888889 |

90. | Ng2007 pragmatic_study 3.555556 |

|-------------------------------------------------------------|

91. | Nitsun1974 intermediate_study 2.888889 |

92. | O’brien1972 intermediate_study 3.444444 |

93. | Odonnell 2003 pragmatic_study 4.444445 |

94. | Patterson2003 intermediate_study 3 |

95. | Patterson2006 intermediate_study 3 |

|-------------------------------------------------------------|

96. | Penades2006 intermediate_study 2.666667 |

97. | Peniston1988 intermediate_study 3.111111 |

98. | Pinto1999 3 int intermediate_study 3.111111 |

99. | Posner1992 pragmatic_study 3.555556 |

100. | Ran2003 intermediate_study 3 |

|-------------------------------------------------------------|

101. | Rector2003 pragmatic_study 3.777778 |

102. | Richardson2007 explanatory_study 2.444444 |

103. | Rohricht2006 pragmatic_study 3.888889 |

104. | Roncone2004 explanatory_study 2.222222 |

105. | Sartory 2005 intermediate_study 3 |

|-------------------------------------------------------------|

106. | Schooler1997 explanatory_study 2.222222 |

107. | Sensky2000 pragmatic_study 4.111111 |

108. | Shin2002 intermediate_study 3.111111 |

109. | Sibitz 2007 intermediate_study 3.333333 |

110. | Silverstein2005 explanatory_study 1.555556 |

|-------------------------------------------------------------|

111. | Smith 1987 intermediate_study 3.333333 |

112. | So2006 pragmatic_study 4.111111 |

113. | Spaulding1999 intermediate_study 3.111111 |

114. | Stanton1984 (gunderson) explanatory_study 1.888889 |

115. | Startup2004 pragmatic_study 3.666667 |

|-------------------------------------------------------------|

116. | Szm1ler2003 pragmatic_study 3.555556 |

117. | Talwar2006 intermediate_study 3.222222 |

118. | Tarrier1998 pragmatic_study 3.888889 |

119. | Trower2004 pragmatic_study 4.111111 |

120. | Tsang 2005 intermediate_study 3 |

|-------------------------------------------------------------|

121. | Turkington2002 pragmatic_study 4.666667 |

122. | Twamley2008 pragmatic_study 3.777778 |

123. | Ucok2006 intermediate_study 2.555556 |

124. | Ulrich2007 intermediate_study 2.888889 |

125. | Valencia2007 check explanatory_study 2.111111 |

|-------------------------------------------------------------|

126. | Valmaggia2005 pragmatic_study 3.777778 |

127. | Vandergaag2002 explanatory_study 2.111111 |

128. | Vaughan1992 pragmatic_study 4 |

129. | Velligan2000 intermediate_study 3 |

130. | Velligan2002 intermediate_study 3.222222 |

|-------------------------------------------------------------|

131. | Velligan2008a intermediate_study 2.888889 |

132. | Velligan2008b intermediate_study 3.333333 |

133. | Vollema 1995 explanatory_study 2.111111 |

134. | Vreeland2006 intermediate_study 3.444444 |

135. | Wykes1999 intermediate_study 3 |

|-------------------------------------------------------------|

136. | Wykes2005 pragmatic_study 4.888889 |

137. | Wykes2007 intermediate_study 3.333333 |

138. | Wykes2007a intermediate_study 3 |

139. | Xiang2006 intermediate_study 3.111111 |

140. | Xiang2007 intermediate_study 3 |

|-------------------------------------------------------------|

141. | Xiong1994 pragmatic_study 3.888889 |

142. | Yang1998 explanatory_study 2.111111 |

143. | Zhang1994 pragmatic_study 3.777778 |

|  |
| --- |
