# Supplementary material for: Are trials of psychological and psychosocial interventions for schizophrenia and psychosis included in the NICE guidelines pragmatic? A systematic review
Source: PLoS One. 2019 Sep 24;14(9):e0222891. doi: 10.1371/journal.pone.0222891 (PMC6759154; doi:10.1371/journal.pone.0222891)
Supplement: S2 Table — (DOCX) [file pone.0222891.s003.docx]

S2_TABLE A RELATIONSHIP BETWEEN PRAGMATISM AND RISK OF BIAS

| Low risk of bias in: | random sequence generation | Allocation concealment | Blinding of participants | Blinding outcome assessment | Incomplete outcome data | Selective reporting | Other bias |
| --- | --- | --- | --- | --- | --- | --- | --- |
| Explanatory studies | 4/23 | 2/23 | 0/23 | 9/23 | 8/23 | 19/23 | 10/23 |
| Pragmatic  studies | 27/48 | 19/48 | 5/48 | 28/48 | 28/48 | 34/48 | 28/48 |
| Intermediate studies | 27/71 | 12/71 | 3/71 | 37/71 | 35/71 | 58/71 | 38/71 |
| Fisher’s exact test: p-value | **0.005** | **0.004** | 0.214 | 0.299 | 0.180 | 0.346 | 0.525 |

S2_TABLE B RELATIONSHIP BETWEEN PRAGMATISM AND OTHER VARIABLES

Replicating the analyses using pragmatism as a three-level categorical variable, the same findings were obtained.

|  | Pragmatic | Intermediate | Explanatory | Test on equality of proportions: p-value |
| --- | --- | --- | --- | --- |
| Sample size (11 missing values) |  |  |  | 0.293 |
| <50 | 13/45 | 29/66 | 11/21 |  |
| 50-100 | 23/45 | 24/66 | 8/21 |  |
| >100 | 9/45 | 13/66 | 2/21 |  |
| Year of publication |  |  |  | 0.223 |
| before 1995 | 7/48 | 18/71 | 8/24 |  |
| 1995-2005 | 27/48 | 32/71 | 13/24 |  |
| after 2005 | 14/48 | 21/71 | 3/24 |  |
| Results (14 missing values) |  |  |  | 0.104 |
| Intervention sign. better | 33/46 | 45/61 | 11/22 |  |
| Intervention not better | 13/46 | 16/61 | 11/22 |  |
| Country (37 missing values) |  |  |  | **<0.001** |
| UK | 19/39 | 6/49 | 1/18 |  |
| North America | 4/39 | 24/49 | 9/18 |  |
| Europe | 9/39 | 9/49 | 3/18 |  |
| Others | 7/39 | 10/49 | 5/18 |  |
