# Supplementary material for: Are trials of psychological and psychosocial interventions for schizophrenia and psychosis included in the NICE guidelines pragmatic? A systematic review
Source: PLoS One. 2019 Sep 24;14(9):e0222891. doi: 10.1371/journal.pone.0222891 (PMC6759154; doi:10.1371/journal.pone.0222891)
Supplement: S1 Fig — S1B FIG Risk of bias of intermediate studies. S1C FIG Risk of bias of explanatory studies. (DOCX) [file pone.0222891.s004.docx]

S1_FIGURE. RISK OF BIAS OF INCLUDED STUDIES


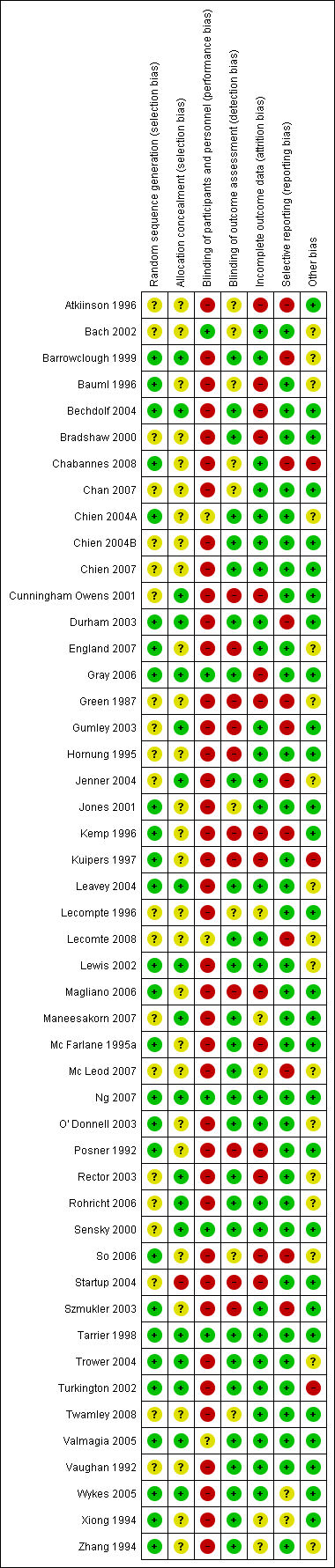


S1A FIGURE. Risk of bias of pragmatic studies


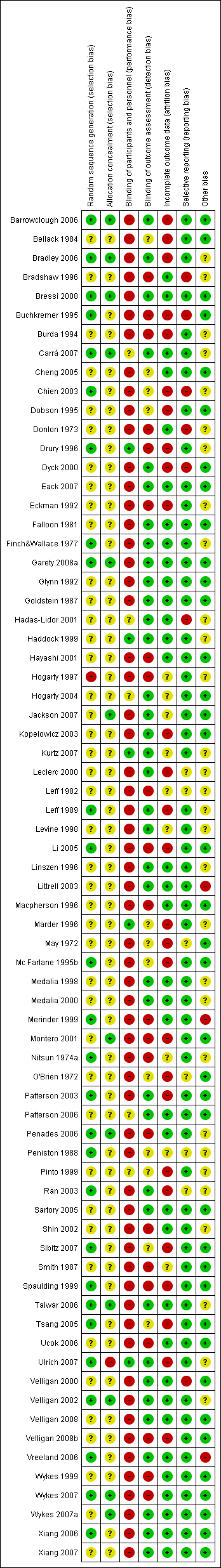


S1B FIGURE. Risk of bias of intermediate studies


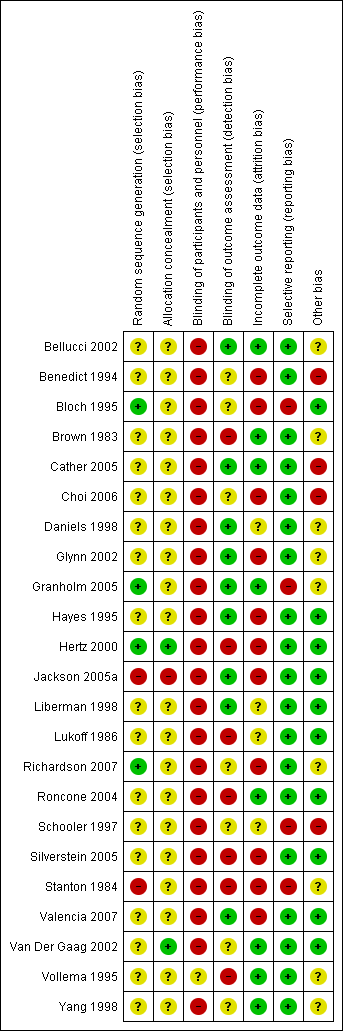


S1C FIGURE. risk of bias of explanatory studies
