# Supplementary material for: Are trials of psychological and psychosocial interventions for schizophrenia and psychosis included in the NICE guidelines pragmatic? A systematic review
Source: PLoS One. 2019 Sep 24;14(9):e0222891. doi: 10.1371/journal.pone.0222891 (PMC6759154; doi:10.1371/journal.pone.0222891)
Supplement: Attachment — Submitted filename: Pone D 19 17507 review.pdf [file pone.0222891.s005.pdf]

PONE-D-19-17507

PRAGMATISM OF NICE GUIDELINES FOR SCHIZOPHRENIA AND PSYCHOSES

Are trials of psychological and psychosocial interventions for schizophrenia and psychosis included in the NICE guidelines pragmatic?

General aspects

It is a review paper in which he seeks arguments for a treatment guide for schizophrenia and other psychoses. There was a careful inclusion criterion of statistical studies and care, although of basic statistics, the results have discussed aspects that gives support to the conclusion.

Here there are some questions:

1) The study presents the results of scientific research. Despite being a systematic review study, the presentation of the results is adequate, being a study of the evaluation of NICE pragmatism from previously published works. This point is important for using the guide.

2. Results reported have not been published elsewhere. Although being a review article, the results were organized in an appropriate way to think about the validity or not of the NICE.

3. A basic statistic was used, but the way the authors clustered the data was adequate.

4. Conclusions are presented in an appropriate fashion and are supported by the data.

5. Regarding the references of the 143 studies, will these studies be in supplemental material?

6. There are two initials that don’t have meanings.

7. It was observed that the author does not explain the meaning of two initials – see pg 7.
